# A survey for ketamine abuse and its relation to the lower urinary tract symptoms in Taiwan

**DOI:** 10.1038/s41598-019-43746-x

**Published:** 2019-05-10

**Authors:** Chiao-Ching Li, Sheng-Tang Wu, Tai-Lung Cha, Guang-Huan Sun, Dah-Shyong Yu, En Meng

**Affiliations:** 1 Division of Urology, Department of Surgery, Tri-Service General Hospital, National Defense Medical Center, Taipei, 11490 Taiwan; 2 Department of Surgery, Kaohsiung Armed Forces General Hospital, Kaohsiung, 80284 Taiwan

**Keywords:** Urological manifestations, Bladder

## Abstract

We aimed to explore the correlation between ketamine abuse and lower urinary tract symptoms (LUTS) and epidemiology of ketamine cystitis. Questionnaire records of ketamine abusers, such as sex, age, and details of using ketamine, including consumption method, amount, duration of ketamine use, and LUTS, were obtained from two private rehabilitation centers. We analyzed these factors and established a severity forecasting module. One hundred and six ketamine abusers completed the questionnaires. LUTS showed an onset time of 24.67 ± 26.36 months among ketamine abusers. Overactive bladder symptom score, international prostate symptom score-storage, interstitial cystitis symptom index, interstitial cystitis problem index, and visual analogue scale score were 5.25 ± 4.43, 5.95 ± 5.72, 10.96 ± 6.66, 9.73 ± 5.82, and 2.55 ± 3.18, respectively. All symptom scores were positively correlated with the duration of ketamine abuse. Ketamine snorting was significantly correlated with all symptom scores compared to smoking. Hydrodistention, intravesical hyaluronic acid instillation, intravesical injection with botulinum toxin, and hyperbaric-oxygen therapy showed better effect than oral treatment. Ketamine can induce severe storage symptoms, such as frequency or nocturia depending on the duration of abuse. Ketamine snorting may cause worse LUTS than smoking. Combining ketamine and other substances may exacerbate LUTS. Intravesical therapy may lead to better outcomes than oral treatment.

## Introduction

Ketamine is a noncompetitive N-methyl-D-aspartic acid receptor complex antagonist and phencyclidine derivative, which was first synthesized in 1962 by Calvin Lee Stevens^1^ and first given to American soldiers during the Vietnam war under FDA approval in 1970^2^. It is currently used for general anesthesia and pain management for chronic neuropathic or malignant diseases^3^.

Ketamine has become a popular recreational drug in nightclubs in Taiwan and other areas, such as Hong Kong and the UK, in the past 10 years^4–6^. Many adolescents use it because of its low price and easy usage. Long-term ketamine abuse may cause severe lower urinary tract symptoms (LUTS), which are very similar to those of interstitial cystitis/bladder pain syndrome (IC/BPS) including increased urinary frequency, urgency, nocturia, intractable dysuria, hematuria, and bladder pain. Since the first report of ketamine-associated urinary tract damage in Canada in 2007^7^, the number of reported cases in recent years has been increasing. Although a study of LUTS in ketamine users reported a prevalence of 26.6% in non-treatment-seeking people^8^, epidemiological data in Taiwan are still lacking.

The treatment for ketamine-associated LUTS is challenging and it resembles that for interstitial cystitis. According to guidelines of the American Urological Association^9,10^, there are six-line options for interstitial cystitis. However, the most important step in the management of ketamine-associated LUTS, which differs from interstitial cystitis, is the discontinuation of ketamine use.

We conducted an in-depth epidemiological study to better understand the correlation between ketamine abuse and LUTS in Taiwan. We hypothesized that various ketamine consumption parameters would be associated with LUTS.

## Results

**Figure 1:**
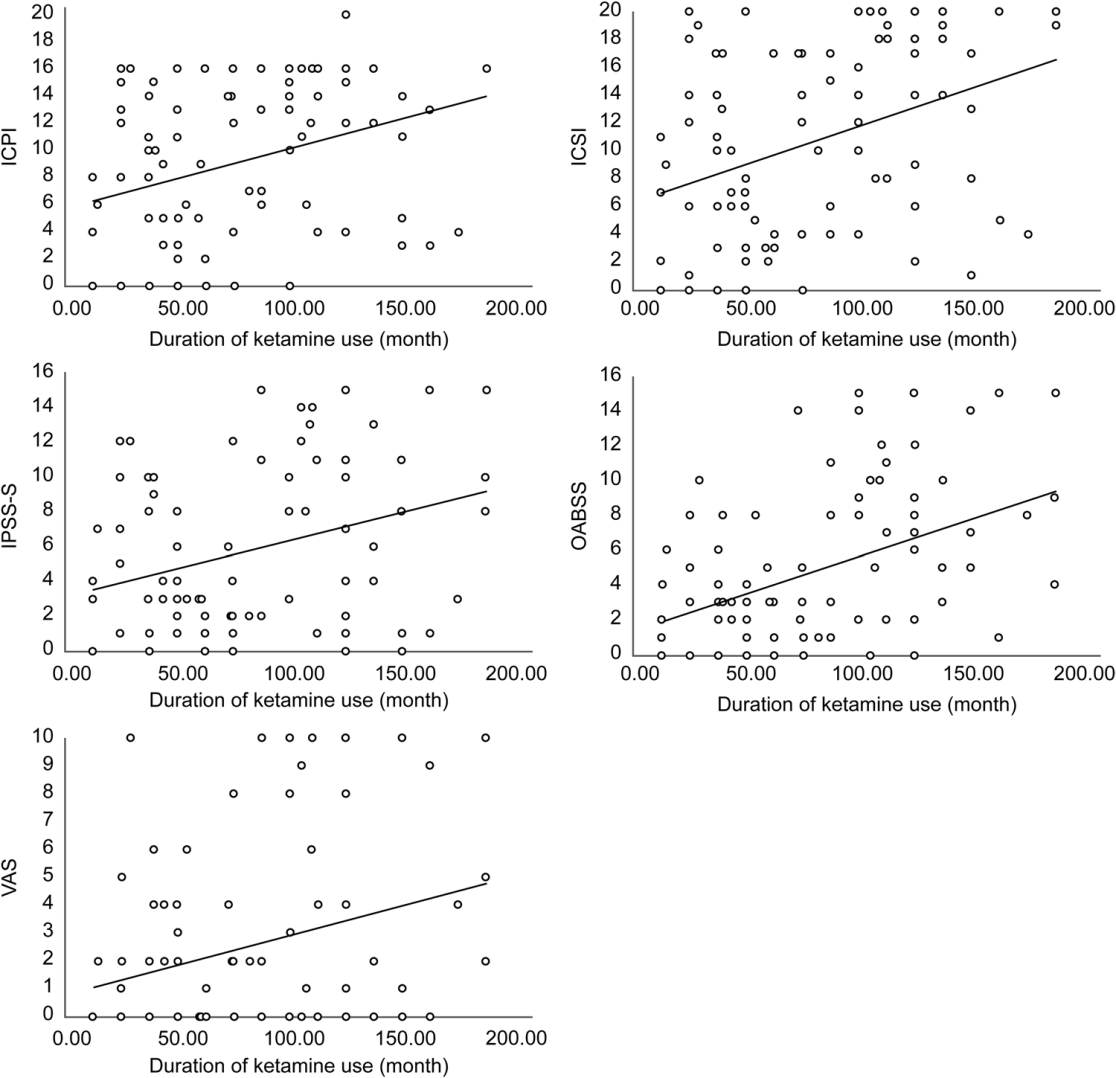
Correlations between symptom scores and duration of ketamine abuse. ICPI: interstitial cystitis problem index; ICSI: interstitial cystitis symptom index; IPSS-S: international prostate symptom score-storage; OABSS: overactive bladder symptom score; VAS: visual analogue scale.

**Figure 2:**
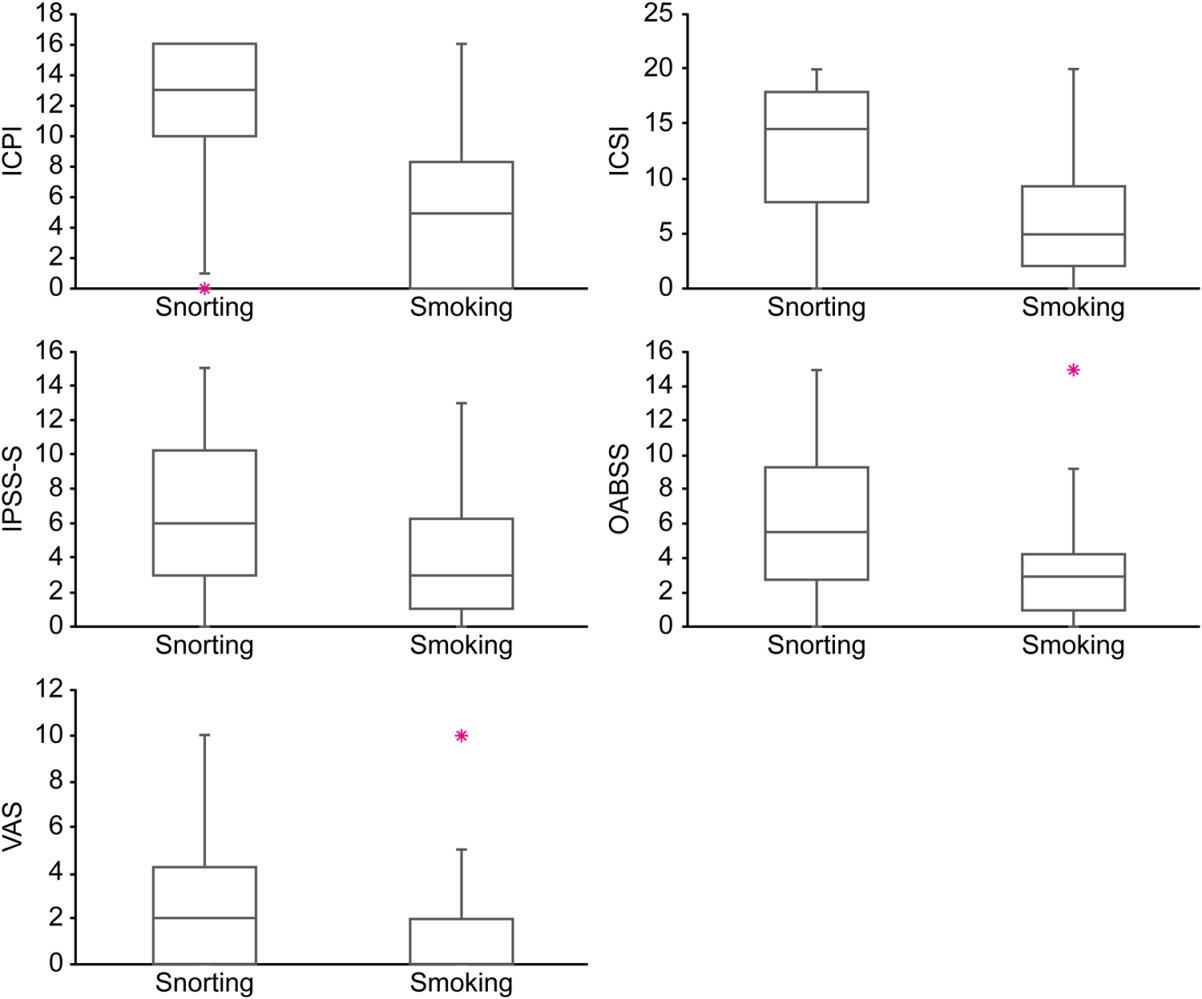
Comparison of symptom scores between ketamine snorting and smoking. ICPI: interstitial cystitis problem index; ICSI: interstitial cystitis symptom index; IPSS-S: international prostate symptom score-storage; OABSS: overactive bladder symptom score; VAS: visual analogue scale.

Ninety-five men and 11 women (24.87 ± 6.44 years old) submitted the questionnaires. The average age of starting ketamine use in this study was 16.68 ± 3.35 years. The duration of ketamine use was 75.45 ± 43.9 months, and cessation interval was 12.79 ± 13.47 months. Eighty-nine (84%) ketamine users developed LUTS after using ketamine for 24.67 ± 26.36 months. Ketamine use methods were snorting (34%), smoking (33%), and both (33%). Ninety-one (92.9%) subjects used ketamine more than once daily. Most of the abusers used no more than 10 gm per day (Table 1). Approximately 80% of ketamine abusers admitted of having a history of polysubstance abuse, although none of them used drugs other than ketamine regularly. There were different combination substances, such as amphetamine, marijuana, morphine, heroin, codeine, flunitrazepam, and ecstasy. Ecstasy was most frequently combined with ketamine (90.5%). Urinary frequency (66, 67.3%), incomplete bladder emptying (65, 67%), and nocturia (60, 61.9%) were the three most common symptoms caused by long-term ketamine use. In addition, the most bothersome symptoms were urinary frequency (55, 56.7%), nocturia (28, 29.3%), and urinary urgency (34, 35.1%). LUTS may develop after using ketamine for about 2 years. LUTS improved after cessation of ketamine use in 81 (83.5%) abusers. Urinary frequency was the first symptom that improved after withdrawal from ketamine abuse. OABSS, IPSS-S, and the scores of ICSI, ICPI, and VAS were 5.25 ± 4.43, 5.95 ± 5.72, 10.96 ± 6.66, 9.73 ± 5.82, and 2.55 ± 3.18, respectively. All the symptom scores were positively correlated with the duration of ketamine abuse (Fig. 1). Ketamine snorting caused significantly more severe symptoms than smoking (*P* < 0.05, Fig. 2). The duration of ketamine use via snorting was longer than that via smoking (91.85 months vs. 56.03 months). OABSS significantly increased with the combined use of ketamine and marijuana (*P* = 0.016). Combined use of ketamine and 3,4-methylenedioxy-methamphetamine (MDMA) significantly increased the ICPI score (*P* = 0.034).

**Table 1:** Characteristics of ketamine abusers.

Variable	Value
Number of ketamine abusers, N	106
Number of men, N (%)	95 (89.6)
Number of women, N (%)	11 (10.4)
Age, years (mean ± SD)	24.87 ± 6.44
Age of first-time ketamine abuse, years	16.68 ± 3.35
Duration of ketamine abuse, months	75.45 ± 43.9
Cessation interval ketamine abuse, months	12.79 ± 13.47
LUTS developed after ketamine abuse, months	24.67 ± 26.36
**Method of ketamine abuse**, **N** (**%)**	
Only snoring	4 (3.8)
Only smoking	4 (3.8)
Mainly snorting	36 (33.3)
Mainly smoking	32 (30.2)
Half-and-half	28 (26.4)
**Frequency of ketamine abuse**, **N** (**%)**	
Several times per day	91 (92.9)
Once daily	3 (3.1)
Once every 2–3 days	2 (2)
Weekly	1 (1)
Sometimes	1 (1)

Fifty-one ketamine abusers (48.1%) did not seek any treatment. All of the scores in the non-treated group were lower than those in the treated group (OABSS: 4.96 ± 4.31 vs. 5.93 ± 4.76, *P* = 0.053; IPSS-S: 5.14 ± 4.36 vs. 7.31 ± 6.71, *P* = 0.0055; ICSI: 9.02 ± 6.33 vs. 13.69 ± 6.18, *P* = 2.77 × 10^−6^; ICPI: 8.07 ± 5.94 vs. 11.96 ± 4.88, *P* = 1.12 × 10^−5^; VAS: 2.03 ± 2.83 vs. 3.31 ± 3.57, *P* = 0.0053). The survey showed that in the treated group, 49 (89.1%) abusers received oral medication for LUTS and 26 (53.1%) of them achieved symptom relief. Other treatments included cystoscopy with hydrodistention, intravesical hyaluronic acid instillation, intravesical injection with Botulinum toxin, and hyperbaric-oxygen therapy, which showed better effect on LUTS than on oral medication (Table 2).

**Table 2:** Treatment for ketamine abuse.

	No. of subjects treated	Response rate
Oral medication	49 (89.1%)	26 (53.1%)
Cystoscopy with hydrodistention	14 (25.4%)	10 (71.4%)
Intravesical hyaluronic acid instillation	7 (12.7%)	5(71.4%)
Intravesical injection with botulinum toxin	2 (3.6%)	2 (100.0%)
Hyperbaric-oxygen therapy	3 (5.5%)	1 (33.3%)

## Discussion

Long-term ketamine abuse may cause severe LUTS and bladder dysfunction. However, epidemiological data of ketamine-associated LUTS in Taiwan remain unknown. Our results show that ketamine abuse can induce severe storage symptoms, such as frequency or nocturia, depending on the duration of ketamine use. The combined use of ketamine and other substances (i.e., marijuana and MDMA) may exacerbate LUTS. Half of the ketamine abusers with LUTS did not seek medical advice.

Ketamine use was found to be predominant in men. This finding is in line with a USA-based report from 2007^11^ and drug misuse declaration from 2009 to 2010 in England. Previous literature has shown that the age of first-time ketamine use was approximately 16 years, which is compatible with our data^12^. Many abusers during this period are easily influenced by their peers.

More than 50% of ketamine abusers had LUTS such as increased urinary frequency and urgency, nocturia, urethral pain, and incomplete emptying, while increased frequency, incomplete emptying, and nocturia were the most bothersome problems of ketamine abusers.

Snorting and smoking are the most frequent ways to use ketamine. Ketamine abusers may chop up ketamine crystals into powder by X-ACTO knife or razor blade, and the powder is then inhaled directly. Once inside the nose and sinuses, the drug is absorbed from the nasal mucosa quickly. The onset time for euphoria is about 3–5 minutes. Our data showed that the frequency of smoking and snorting was nearly equal; however, ketamine snorting caused more severe LUTS (Fig. 2). This is likely due to a higher amount of ketamine entering the circulation via direct snorting. In addition, the duration of ketamine use via snorting was longer than that via smoking, which might be another reason why ketamine snorting induced more severe LUTS than smoking.

All the symptom scores were positively correlated with the duration of ketamine abuse, implying that more the contact time with ketamine, the more obvious LUTS and bladder pain become. It generally takes about 2 years to develop LUTS in ketamine abusers. In our study, OABSS significantly increased with the combined use of ketamine and marijuana (*P* = 0.016). Cannabinoids are the active components of marijuana, and select cannabinoid receptors, CB1 and CB2, have been identified in the human detrusor and urothelium^13^. Activation of these receptors could modulate bladder afferent activity and the micturition reflex^14^. In patients with painful bladder syndrome and idiopathic detrusor overactivity, the density of CB1 expression is enhanced in line with afferent sprouting^15^. This might explain the worse storage symptoms in the abusers who combined the use of ketamine and marijuana.

Combined use of ketamine and MDMA significantly increased the ICPI score (*P* = 0.034). The major mechanism of MDMA is central stimulation and a rapid liberation of noradrenaline from peripheral adrenergic terminals. Ketamine may exacerbate MDMA-induced central dopamine toxicity. Whether MDMA worsens LUTS via central stimulation is to be clarified by further research.

Ketamine-associated LUTS was found to be under-treated. About 50% of ketamine abusers with LUTS in this survey did not seek medical advice. All of the symptom scores in the non-treated group were lower than those in the treated group. These data suggested that half of ketamine abusers with LUTS experienced mild symptoms, which did not affect their daily life. For those who received treatment, oral medication was the most common therapy, but the response rate was only 53%. Intravesical therapy, such as hydrodistention, hyaluronic acid instillation, and Botulinum toxin injection, seemed to have a better effect. This result is compatible with the treatment outcome of interstitial cystitis^16^. Since there are only a few reported case series, a standard treatment plan for ketamine cystitis has not been established. In the current study, intravesical botulinum injection had the highest response rate among different kinds of intravesical therapy; however, the patient number was small. This result is compatible with that of a previous study^17^. Nonetheless, whether this should be considered as a primary therapy for ketamine cystitis needs further investigation.

On March 5, 2019, the US Food and Drug Administration has approved esketamine (SpravatoTM) for treatment-resistant depression^18^. Esketamine is a nasal solution available as a nasal spray. Although its recommended daily dosage is low, there are concerns whether its long-term usage may cause lower urinary tract problems, such as urgency, painful sensation, and irregular frequency. The amount of published data on the long-term safety of ketamine treatment in mood disorders is extremely limited and are mostly case series. Therefore, a long-term, cohort study is warranted.

The main limitation of this study is that there was only subjective assessment and a lack of objective assessment. There was also lack of control and objective assessment in the study. The results may have sex bias as the study had few female participants. Details of oral medication could not be verified; thus, we were not able to determine which medicine was effective to relieve LUTS caused by ketamine abuse.

## Conclusions

Ketamine abuse can cause voiding dysfunction. The severity of storage symptoms is correlated with the duration of ketamine use. Ketamine snorting may cause worse LUTS than smoking. The combined use of ketamine and other substances, such as marijuana and MDMA, may exacerbate LUTS. Intravesical therapy may produce better outcomes than oral medical treatment. With this model as a platform, further prospective studies are warranted to investigate the appropriate choice of treatment for this new clinical entity.

## Materials and Methods

The self-administered questionnaires were employed in a cross-sectional design and ethical approval by the Institutional Review Board of Tri-Service General Hospital was obtained for data collection and analysis. All procedures performed in studies involving human participants were in accordance with the ethical standards of the institutional and/or national research committee and with the 1964 Helsinki declaration and its later amendments or comparable ethical standards. Informed consent was obtained from all individual participants included in the study.

Before answering the questionnaires, all participants were provided a verbal explanation about the contents of questionnaires and they provided informed consent. The scales included overactive bladder symptom score (OABSS), International Prostate Symptom Score-storage (IPSS-S), interstitial cystitis symptom index (ICSI), interstitial cystitis problem index (ICPI), and visual analogue scale (VAS). We gathered these data from two private rehabilitation centers in Taiwan between January 2015 and December 2015. Ninety-five men and 11 women were enrolled in the study. The survey contained information on sex, age, age when ketamine use started, and how they used ketamine (i.e., the amount and frequency of ketamine use). Details of their LUTS and treatment were also recorded. Ketamine users were also asked whether they were using a combination of ketamine and other common substances, such as amphetamine, marijuana, morphine, heroin, codeine, flunitrazepam, or ecstasy. The treatments that the subjects had received, such as oral medication, hydrodistention via cystoscopy, intravesical therapy, or hyperbaric-oxygen therapy, were verified. The symptom that improved first and the most bothersome symptom were also recorded. Data processing and statistical analysis were performed using SPSS Version 17.0. (SPSS Inc. Chicago) and STATISTICA 7.

## Data Availability

The datasets generated during and/or analysed during the current study are available from the corresponding author on reasonable request.
